# Supplementary material for: The causal relationship between the human gut microbiota and pyogenic arthritis: a Mendelian randomization study
Source: Front Cell Infect Microbiol. 2024 Nov 26;14:1452480. doi: 10.3389/fcimb.2024.1452480 (PMC11629706; doi:10.3389/fcimb.2024.1452480)
Supplement: Supplementary file 1 [file DataSheet1.zip › mendelian test/ebi-a-GCST90027802.csv_forest.pdf]

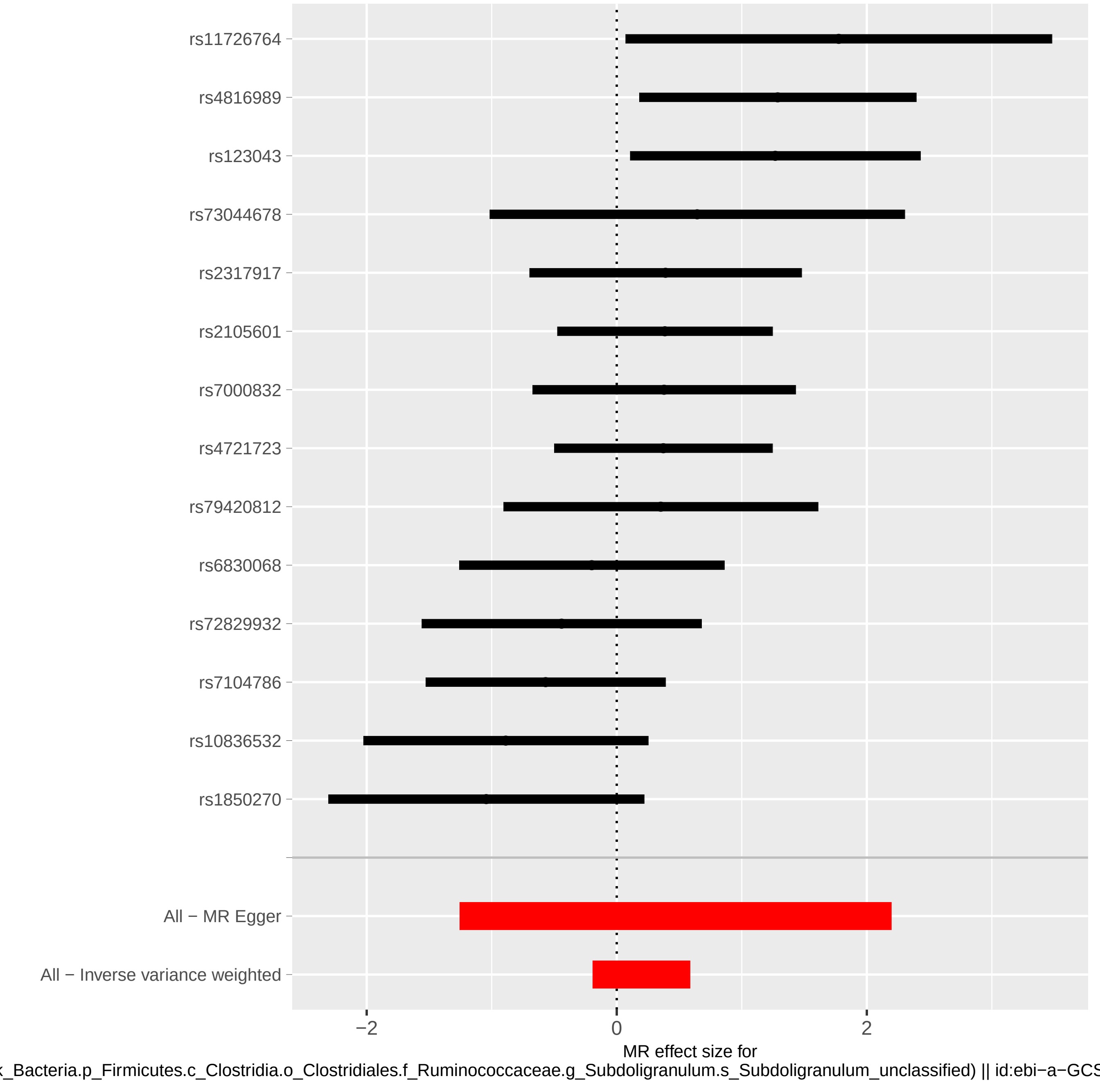
rs11726764
rs4816989
rs123043
rs73044678
rs2317917
rs2105601
rs7000832
rs4721723
rs79420812
rs6830068
rs72829932
rs7104786
rs10836532
rs1850270
All − MR Egger
All − Inverse variance weighted
−2
0
2
MR effect size for
k_Bacteria.p_Firmicutes.c_Clostridia.o_Clostridiales.f_Ruminococcaceae.g_Subdoligranulum.s_Subdoligranulum_unclassified) || id:ebi−a−GCS
